# Influence of ion beam current on the structural, optical, and mechanical properties of TiO_2_ coatings: ion beam-assisted vs conventional electron beam evaporation

**DOI:** 10.3762/bjnano.16.81

**Published:** 2025-07-14

**Authors:** Agata Obstarczyk, Urszula Wawrzaszek

**Affiliations:** 1 Wroclaw University of Science & Technology, Faculty of Electronics, Photonics and Microsystems, Janiszewskiego 11/17, 50-372 Wroclaw, Poland

**Keywords:** electron beam evaporation, ion beam-assisted deposition, mechanical properties, nanocrystalline anatase, optical properties, TiO_2_ coatings

## Abstract

In this paper, comparative studies of selected properties of titanium dioxide (TiO_2_) coatings deposited using electron beam evaporation (EBE) and ion beam-assisted deposition (IBAD) are presented. Post-process annealing at 800 °C was also conducted to examine its impact on the properties of the prepared coatings. After annealing at 800 °C, a transition from amorphous to the anatase phase occurred for all coatings. In particular, an increase in ion beam current led to a reduction in crystallite size by approximately 30% compared to coatings prepared by conventional EBE process. The average anatase crystallite size for annealed films was in the range of 30.8 to 43.5 nm. A detailed SEM analysis of surface morphology and cross sections revealed that the TiO_2_ films prepared by IBAD had smaller, rounded grains and were denser compared to those deposited by EBE. Optical properties showed high transparency of 77–83% in the visible wavelength range for all as-prepared thin films. However, annealing caused a decrease of the transparency level by 32% for films deposited by EBE, while for films from the IBAD process the decrease was less than 10%. The use of an ion gun increased the hardness of the TiO_2_ films from 2.4 to 3.5 GPa (*I*_ibg_ = 4 A). Although a similar relationship was observed for coatings after annealing, hardness values were lower than for as-deposited coatings. The most notable differences were observed in the abrasion tests, where the IBAD process significantly enhanced the abrasion resistance of the coatings. This research highlights the potential of IBAD to prepare dense, adhesive, and durable TiO_2_ coatings with improved optical and mechanical properties, suitable for applications requiring enhanced wear resistance.

## Introduction

One of the commonly used methods for the deposition of various materials for thin film optical coatings, is electron beam evaporation (EBE) [1–5]. Today, in many applications, including medicine, telecommunications, optoelectronics, photovoltaics, the requirements for optical coatings are very high. The electron beam evaporation process uses the high kinetic energy of an electron beam to generate the thermal energy required to melt and then evaporate the source material to deposit it on the substrate [4]. Evaporation is an attractive deposition technique because of its many advantages including low manufacturing cost, high deposition rate, and the possibility to coat large surface areas [3,5–6]. However, the quality of the deposited coatings is relatively poor compared to, for example, magnetron sputtering, which guarantees the production of thin films with good coating adhesion to substrates and a densely packed structure. Also, the deposition rate is low, which affects the high residual stress of the coating [7]. In order to improve the properties of vapor-deposited coatings, it is necessary to increase the total energy of the particles reaching the substrates. In practice, additional heating of the substrates, reducing the pressure in the working chamber, applying additional electrical bias to the substrates, or using ion beam assistance are used. All of these methods lead to an increase in the total energy of the nucleating particles on the substrate.

Ion beam-assisted deposition (IBAD), which is the bombardment of a thin film with a beam of energetic particles during deposition, is an excellent technique for modifying the microstructure and optical, mechanical, and tribological properties of thin film coatings [8–11]. This energetic process offers many possibilities and is easily implemented using conventional equipment [8]. The additional energy supplied to the deposited atoms (typically 60–180 eV compared to the energy of simply evaporated particles of not more than 0.1 eV) results in atomic displacements in the growing coating and enhanced surface atom migration. This can result in much better adhesion of the films to the substrate [11]. In addition, the advantage of using additional ion beam assistance in the EBE processes is an increased packing density of the coatings, making them more resistant to moisture [8,12]. Moreover, the IBAD technique has become one of the methods for producing high-quality optical thin film coatings. According to [13–14], IBAD support of the electron beam evaporation process affects the properties thin films like formation of new phases, modification of residual stress, elimination of the columnar-like character of the structure, and the improvement of stability and homogeneity of the coatings. In addition, it is possible to modify the microstructure, resulting in dense, nearly stoichiometric films that are much more resistant to temperature and humidity changes than films deposited in the conventional EBE process [4,9–12].

Electron beam evaporation is very effective for preparing transparent titanium dioxide thin films, such as optical coatings for a large variety of applications. Due to its desirable structural, optical, and electrical properties, high thermal and chemical stability, relatively low price, and good availability, TiO_2_ in the form of thin films is now widely used in the development of gas sensors, photodetectors, solar cells, memristors, and photocatalysts [1,12,15–17]. The area of application of titanium dioxide is also related to the crystal structure in which it occurs, that is, brookite, anatase, or rutile [16–20]. The rutile phase is the most stable structure of TiO_2_, while anatase is a metastable phase. Recently, the anatase phase of TiO_2_ has been particularly used in the production of solar cells and optical coatings. According to [17–18], titanium dioxide in the anatase phase, compared to other phases, is particularly favorable for electrochromic applications due to its efficient ion transport and relatively open configuration of octahedral units. These two phases of titanium dioxide differ significantly in their properties in terms of electronic applications [21]. According to [22], amorphous or anatase phases are desirable for optical thin films because of the materials’ isotropic properties with low extinction coefficient.

Considering the above advantages, in this paper, titanium dioxide thin films were prepared using electron beam evaporation and ion beam-assisted deposition with different values of ion beam current. Additionally, post-process annealing at 800 °C was applied to the fabricated samples in order to study its influence on the coatings’ properties. The literature lacks extensive studies of titanium dioxide thin films deposited by electron beam evaporation with ion-assisted deposition followed by high-temperature annealing. This highlights the unique and valuable contribution of our research, as such comprehensive studies are not currently available.

## Experimental

Titanium dioxide optical thin film coatings were prepared by electron beam evaporation with and without additional ion beam assistance. Moreover, all as-deposited films were additionally modified by post-process annealing at 800 °C. As a source material, titanium pentoxide (Ti_3_O_5_) pellets with a purity of 99.99 atom % (from K.J.Lesker) were used, which were evaporated from a molybdenum crucible. Diffusion and a rotary backing pumps enabled a base pressure in the vacuum chamber below 1.5·10^−5^ mbar. The operating pressure in the IBAD process was kept below 1.7·10^−3^ mbar, and the thin film coatings were deposited with an additional oxygen gas flow of 100 sccm. The ion beam gun (Advanced Energy) DC current (*I*_ibg_) and voltage were equal to 3 and 4 A and 80 and 90 V, respectively (samples denoted as S0A, S3A and S4A). Each process lasted 60 min, and the thicknesses of the prepared films, measured with an optical profilometer (Talysurf CCI Lite), were 350, 300, and 200 nm for samples S0A, S3A, and S4A, respectively. The deposition parameters are shown in Table 1. Like in our previous reports [23–24], additional post-process modification of as-prepared thin films was carried out, that is, annealing at 800 °C (Nabertherm tubular furnace). The post-process annealing was carried out for 2 h in ambient atmosphere with a heating ramp of 200 K·h^−1^, without the use of refrigerants.

**Table 1:** Deposition parameters in EBE and IBAD processes.

	EBE	IBAD

pellet material	Ti_3_O_5_ ( 99.99 atom %)	
substrate material	SiO_2_, Si, TiAlV	
base pressure	1.5·10^−5^ mbar	
substrate temperature	unheated (<50 °C)	
distance from evaporation source to substrate	500 mm	
deposition time	60 min	
rotation of the substrates	3 rpm	
additional O_2_ gas flow	100 sscm	
operating pressure	(1.2–1.7)·10^−3^ mbar	
ion beam gun current, *I*_ibg_	—	3 A, 4 A
ion beam gun voltage	—	80 V, 90 V

The effect of ion beam-assisted deposition and additional post-process modification of TiO_2_ coatings was investigated in detail. XRD measurements (PANalytical Empyrean PIXel3D), Raman spectroscopy (Thermo Fisher Scientific Raman Microscope), and SEM imaging (FEI Nova NanoSEM 230) were carried out to evaluate the structural properties and morphology of the prepared coatings. The optical characteristics of the prepared films were investigated with the use of an Ocean Optics QE65000 spectrophotometer in the wavelength range of 250–1000 nm. As in our previous work [25], nanoindentation and wear resistance tests were used to determine the mechanical and tribological properties of the prepared coatings. The hardness of the prepared coatings was determined by nanoindentation using a CSM Instruments nanoindenter with a diamond Vickers tip. For the determination of the hardness from the load–displacement data, the Oliver and Pharr method was used [26].

Wear resistance was investigated using the Summers Optical’s Lens Coating Hardness Test Kit. In our previous work [27], the steel wool test was described and carried out according to the well-acknowledged standard [28] and literature reports [29]. After the abrasion tests, the surface of thin film coatings was examined in detail by optical microscopy (Olympus BX51) and optical profilometry (TalySurf CCI Lite Taylor Hobson).

## Results and Discussion

**Figure 1:**
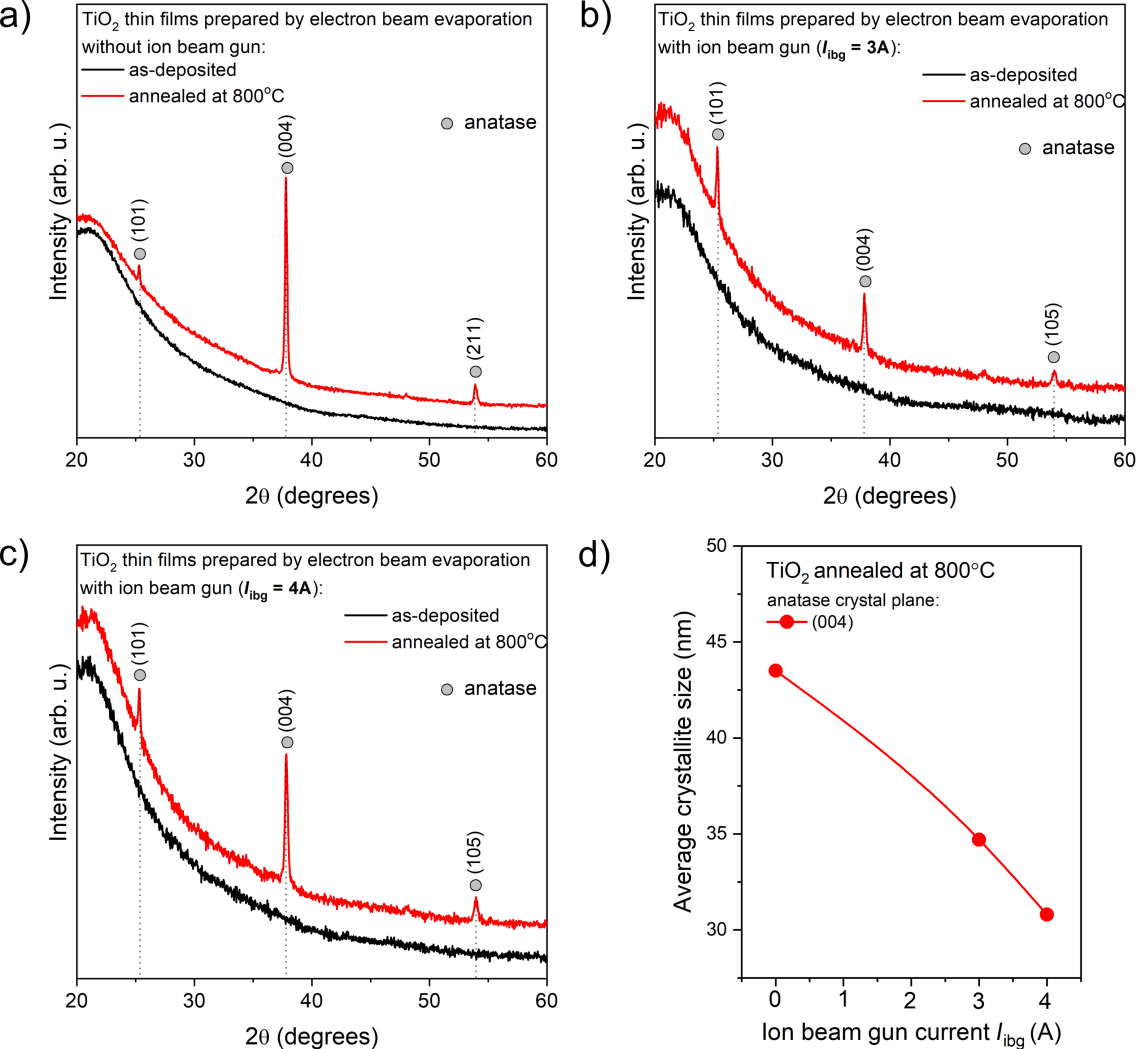
Comparison of XRD patterns of TiO_2_ thin films deposited by (a) EBE and (b, c) IBAD before and after additional post-process annealing at 800 °C, respectively. (d) Dependence of the average crystallite size on *I*_ibg_ for the annealed TiO_2_ thin films.

Figure 1 shows a comparison of XRD patterns of as-deposited and annealed TiO_2_ thin films prepared by EBE and IBAD. All thin films changed their structure from amorphous to anatase after annealing. Mergel et al. [30] and Lu et al. [3] showed that TiO_2_ films prepared by the EBE method, due to the low substrate temperature (below 300 °C), are always amorphous. Oliver et al. [31] has also indicated that TiO_2_ thin films obtained from conventional electron beam evaporation typically exhibit a porous structure. The use of ion beam-assisted deposition in the EBE process had no effect on the structure of the as-prepared TiO_2_ coatings (Figure 1). Therefore, post-deposition annealing becomes one of the key pathways in controlling the structure, morphology, and photocatalytic activity of TiO_2_ films [3].

After post-process annealing, all thin films exhibited peaks related to the (101) and (004) planes of anatase, for which the crystallites sizes were also determined. The crystallite size was calculated using MDI Jade 5.0 software employing the Scherrer equation [32]. Thin film coatings deposited by EBE method had crystallites with an average size of 43.5 nm calculated from the (004) plane. In turn, increasing the ion gun current from 3 to 4 A caused a decrease in crystallite size from 34.7 to 30.8 nm after annealing (Figure 1d). The results of the structure analysis are shown in Table 2.

**Table 2:** XRD analysis of TiO_2_ thin films deposited by EBE without and with IBAD after additional post-process annealing at 800 °C.^a^

Annealed thin films	Phase	Crystal plane	Crystallite size (nm)	*f*	d (nm)	Δ*d* (%)	Type of stress

PDF No. 21-1272 TiO_2_	anatase	(004)	—	—	0.2378	—	—
S0A			43.5	0.834	0.23776	−0.02	compressive
S3A			34.7	0.343	0.23776	−0.02	compressive
S4A			30.8	0.605	0.23767	−0.05	compressive

^a^*f*: degree of preferential orientation; *d*: measured interplanar distance; Δ*d*: percentage change of the measured interplanar distance as compared to the standard *d*_PDF_.

Based on the XRD patterns, there is no evidence for the occurrence of TiO_2_ with the rutile phase, as its specific peaks were not observed. It can be suspected that TiO_2_ deposited by the EBE method exhibits a preference for amorphous phase growth. It seems that the way to obtain crystalline TiO_2_ coatings is through post-process modification, such as annealing at elevated temperatures, which induces titanium and oxygen atoms to reorganize and form a regular crystal structure.

To determine the degree of the preferential orientation, the texture factor (*f*) was estimated using the Lotgering method. A value of *f* = 0 indicates a random orientation, while *f* = 1 testifies the perfect orientation for the calculated plane [33–35]. For the annealed TiO_2_ thin films, the preferred orientation was calculated for the (004) lattice plane. The highest value of texture factor was determined for the film prepared without IBAD and was equal to 0.834. In the case of films prepared with additional ion beam assistance, the Lotgering factor was 0.343 and 0.605 for ion beam gun currents of 3 and 4 A, respectively.

The type of stress occurring in the annealed TiO_2_ thin films was determined based on the parameter Δ*d* [36–37]. This parameter represents the relative difference between the measured interplanar distance and the standard value. When Δ*d* is lower than zero, compressive stress occurs; when Δ*d* is higher than zero, tensile stress is observed. After post-process annealing, a slight shift in the measured diffraction peaks toward higher angles was observed, indicating compressive stress with values from −0.05 to −0.02. The compressive stress after annealing was negligible, which can be attributed to the improved hardness and densification of the IBAD films, as confirmed by microstructural, nanoindentation, and wear resistance analyses. Mitigation or control of residual stress can be achieved by optimizing deposition parameters such as substrate temperature, deposition rate, substrate biasing, and ion beam gun settings. Moreover, post-process annealing can lead to a reduction in thermal stress due to the difference in thermal expansion coefficients between the film and the substrate and can also affect internal stress by changing the microstructure of the film.

**Figure 2:**
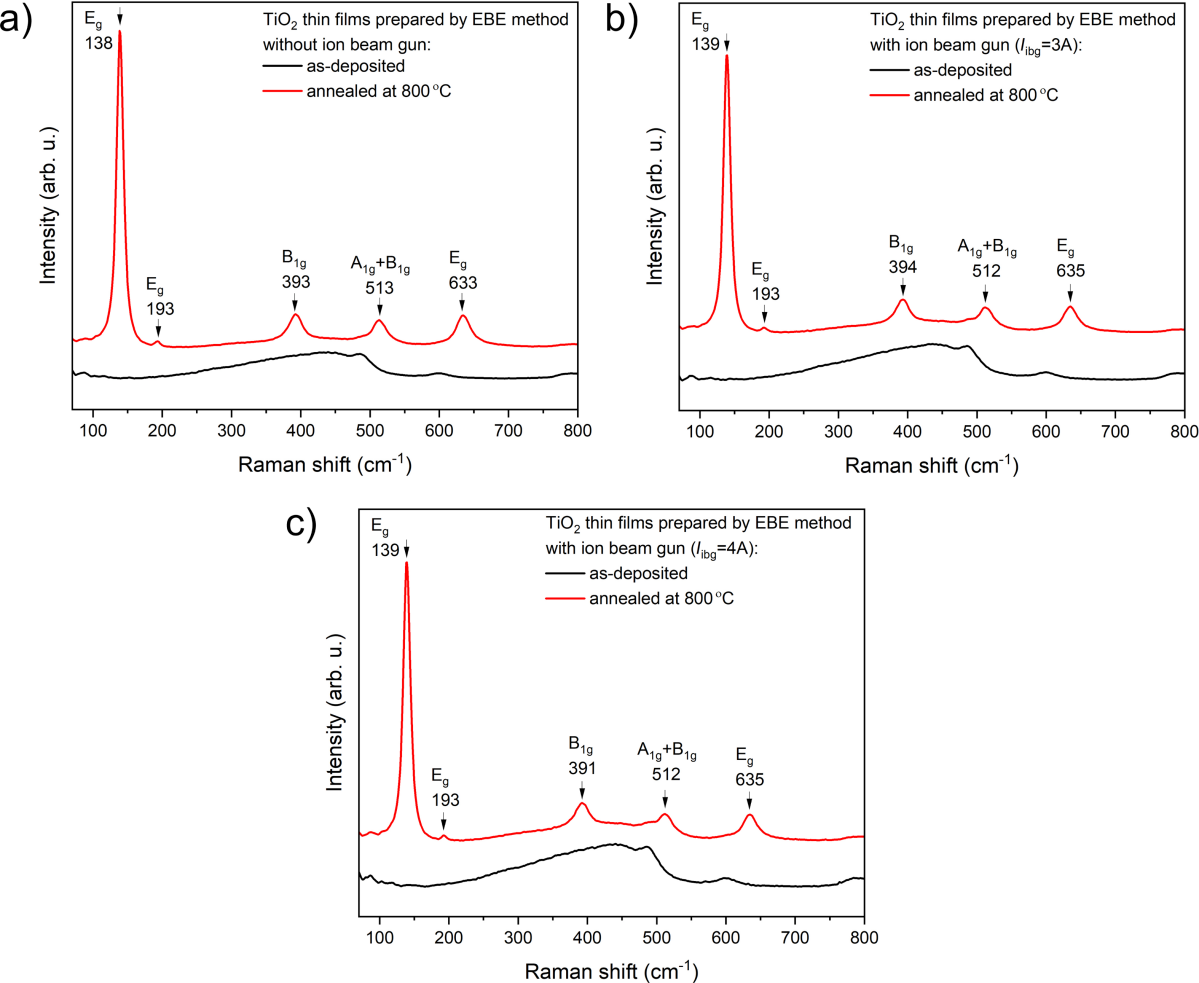
Raman spectra of TiO_2_ thin films deposited by (a) EBE and (b, c) IBAD before and after additional post-process annealing at 800 °C, respectively.

Figure 2 shows Raman spectra of the TiO_2_ films prepared by EBE and IBAD methods. The microstructure of the prepared thin films was not affected by the additional use of the ion beam gun since only the amorphous phase was observed for all coatings. The Raman results confirm the phase change from amorphous to crystalline after annealing at 800 °C, in agreement with the XRD studies. The results obtained by Raman spectroscopy for the annealed films are consistent with the reference values for the anatase phase [38–39]. The presence of characteristic Raman peaks observed at approximately 139, 193, 393, 512, and 635 cm^−1^ confirms the occurrence of the anatase phase in the annealed coatings (Figure 2) [40–41].

**Figure 3:**
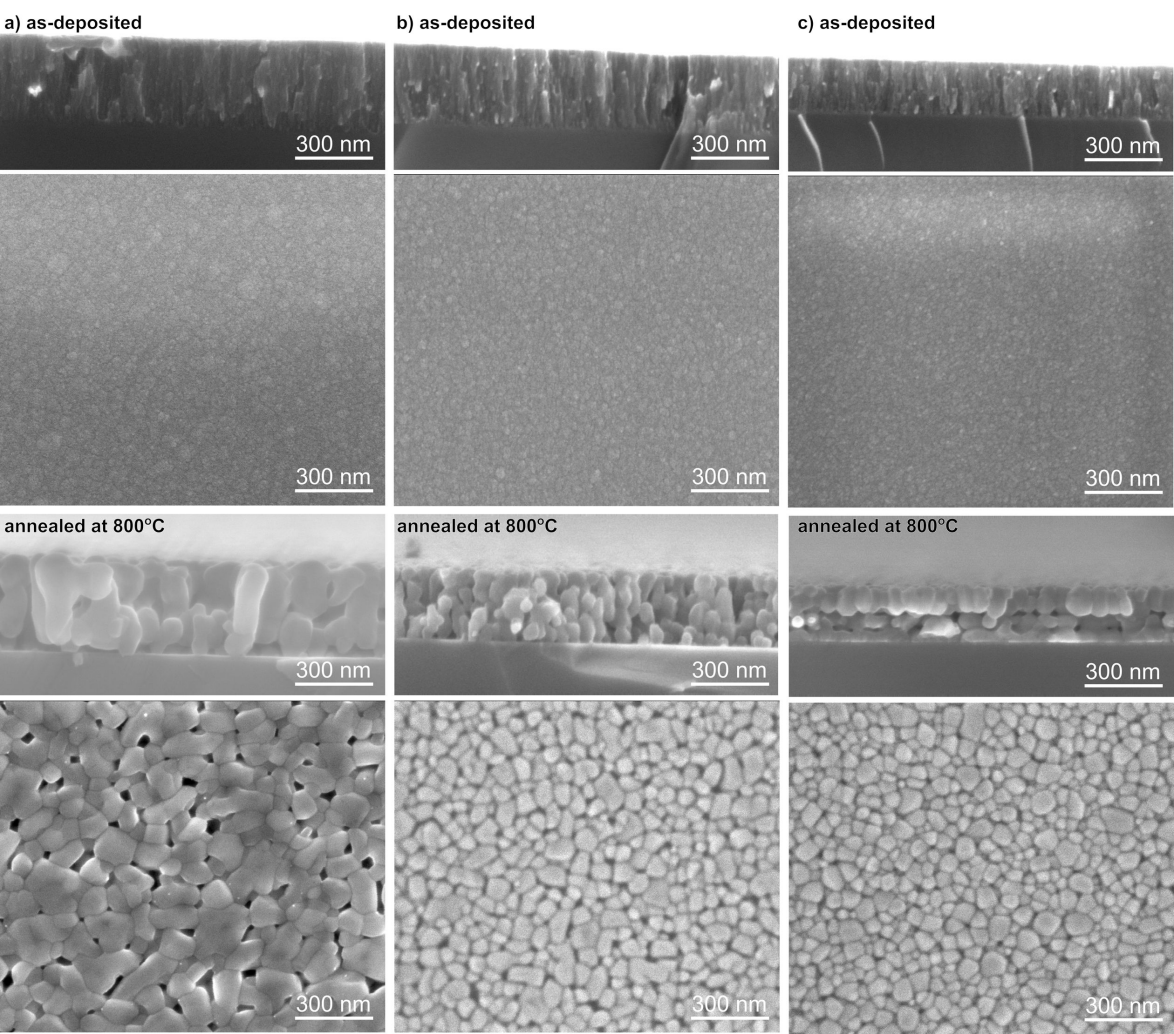
Scanning electron microscopy images of TiO_2_ thin films before and after post-process annealing at 800 °C deposited using (a) EBE and IBAD with (b) *I*_ibg_ = 3 A and (c) *I*_ibg_ = 4 A.

Figure 3 presents SEM images of the surface and cross sections of TiO_2_ thin films before and after post-process annealing. In the case of as-prepared TiO_2_ coatings, there are no significant differences between the surface morphology for films deposited without and with ion gun. SEM images showed smooth surfaces composed of very small grains with columnar-like character. Post-process annealing caused a significant change of the surface and cross-section morphology. After thermal modification, the surface morphology of the prepared coatings without additional ion bombardment showed very large grains with an average size of approximately 100 nm, which formed agglomerates with visible voids between them (Figure 3 A). Additionally, the cross-section image showed that the annealed film was composed of big, elongated grains with a length from 160 to 350 nm (Figure 3a). It is worth noting that the coatings had a milky color. This was probably the result of the size of grains, which could lead to a significant scattering of light. In contrast, the use of the ion gun resulted in much smaller, rounded grains and denser coatings. In the case of films deposited with an additional ion gun current of 3 A, the grain size was about 58 nm, while increasing the current to 4 A reduced the grain size again to an average value of 45 nm. Post-process annealing altered the cross-section morphology of the titanium dioxide coating deposited with *I*_ibg_ = 3 A (Figure 3b), changing its fibrous structure to a coarse-grained one. The width of these elongated grains ranged from 60 to 120 nm. Contrary to this, the cross-section image (Figure 3c) of the TiO_2_ coating deposited with *I*_ibg_ = 4 A revealed the formation of a bilayer, indicating the separation of grains into two distinct rows or layers. The width of the elongated grains in this case ranged from 90 to 115 nm. Yang et al. [6] mentioned that compared to conventional electron beam evaporation, ion bombardment provides particles deposited on a substrate with more energy, contributing to the formation of a denser structure. These considerations were also confirmed by XRD and SEM studies (Figure 1 and Figure 3, respectively), indicating the presence of a finely crystalline and dense TiO_2_ structure.

**Figure 4:**
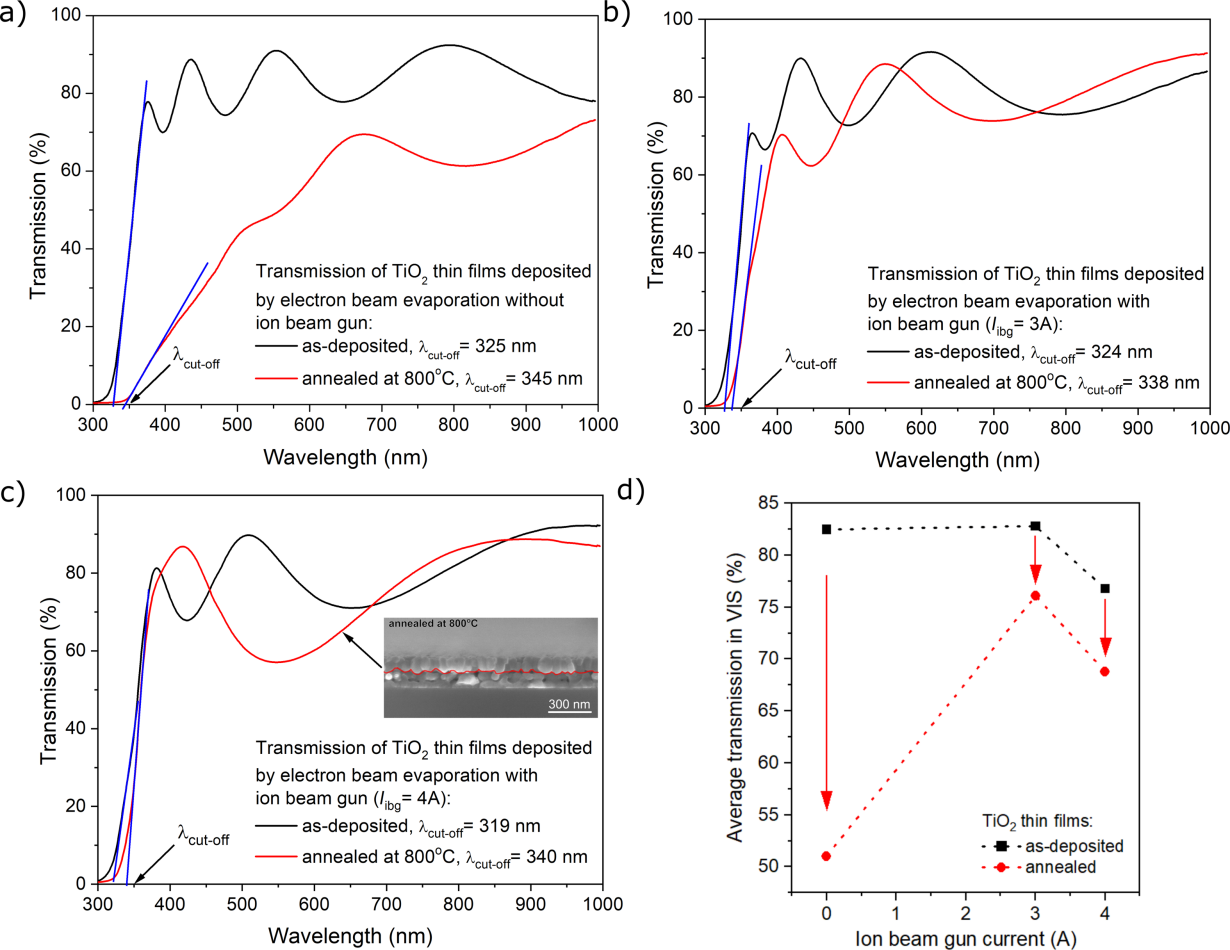
Transmittance spectra of TiO_2_ thin films before and after post-process annealing at 800 °C deposited (a) without and with IBAD with (b) *I*_ibg_ = 3 A and (c) *I*_ibg_ = 4 A. (d) Dependence of the average transmission on the ion beam gun current.

Based on the measured light transmission spectra (Figure 4), all as-deposited coatings had high transparency, ranging from 77% to 83%. The average transparency of the thin films in the visible wavelength range was calculated taking into account the integral of the transmission coefficient in the wavelength range of 380 to 750 nm. The light transmission level for TiO_2_ films deposited by the EBE method was 82.5%, at average, while for films prepared with *I*_ibg_ of 3 A and 4 A, the transparency was 82.9% and 76.8%, respectively (Figure 4d). For a coating deposited using the conventional EBE method, annealing resulted in a decrease in transmission to 51% compared to the as-deposited coating (Figure 4d). In contrast, in the case of thin film coatings deposited with the assistance of the ion gun, annealing did not affect their light transmission coefficients significantly. The transparency level after post-process annealing was equal 76.1% and 68.8%, for TiO_2_ prepared with *I*_ibg_ of 3 and 4 A, respectively (Figure 4d). The cut-off wavelengths of the annealed thin films were redshifted by about 14–21 nm.

**Figure 5:**
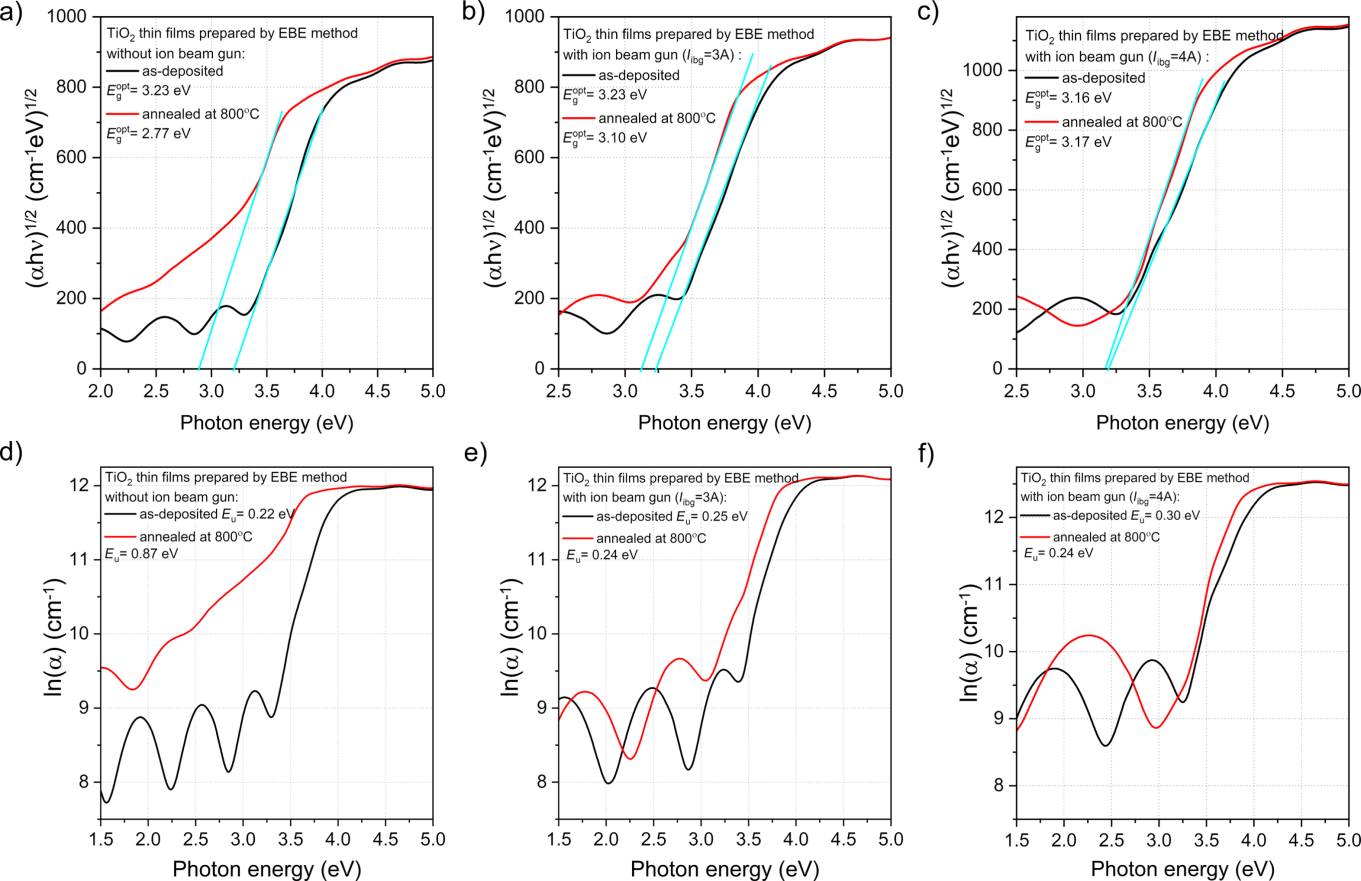
(a–c) Optical bandgap energy and (d–f) Urbach energy as function of the photon energy.

Measurements of spectral characteristics of light transmission provide information not only about the amount of light transmitted through the coating, but also information about the band structure of the materials from which it is made of. Therefore, to perform a comprehensive analysis of the band structure of the prepared titania films, optical bandgap energy (*E*_g_^opt^) and Urbach energy (*E*_u_) were analyzed. The value of *E*_g_^opt^ was calculated by extrapolating the linear portion of the curves [42] based on the plot of (α*h*ν)^1/2^ as a function of photon energy (*h*ν) (Figure 5). Based on the literature [43–45], titanium dioxide in the anatase phase is an indirect-bandgap semiconductor. In the case of the films deposited using the conventional EBE method and an additional *I*_ibg_ of 3 A, the optical bandgap energy was equal to 3.23 eV, while increasing *I*_ibg_ to 4 A led to a slight decrease of *E*_g_^opt^ to 3.16 eV. After post-process annealing, the value of the optical bandgap energy decreased for the film deposited without any additional assistance of the ion gun and was equal to 2.77 eV. In contrast, for films prepared with additional *I*_ibg_ of 3 and 4 A, the *E*_g_^opt^ values were equal to 3.10 and 3.17 eV, respectively. For the annealed TiO_2_ film prepared using the conventional EBE process, the significant change in *E*_g_ could be a result of a considerable increase in grain size after annealing. In contrast, such notable differences were not observed for films deposited by IBAD. In the case of annealed films deposited with *I*_ibg_ = 3 A, a decrease of the bandgap from 3.23 to 3.10 eV was observed. According to Dejam et al. [46], increasing the annealing temperature improved the quality of the crystallites, which reduced the localized states and traps in the thin films, leading to a decrease in the bandgap. However, as can be observed (Figure 5c), the values of optical bandgap energy for as-deposited and annealed coatings prepared by IBAD with an additional *I*_ibg_ of 4 A were equal to 3.16 and 3.17 eV, respectively. According to our previous work [47], for both as-prepared and annealed TiO_2_ coatings (annealed at temperatures ranging from 200 to 600 °C), the optical bandgap (*E*_g_^opt^) was ca. 3.20 eV. A decrease in *E*_g_^opt^ to 2.77 eV was observed for TiO_2_ annealed at 800 °C. We also showed [23] that as-deposited TiO_2_ films were amorphous and *E*_g_^opt^ was equal to 3.29 eV. The thin films in both studies [23,47] were prepared using the conventional EBE method. The results presented in [23,47] also provide evidence that the deposited by the EBE process were reproducible due to very similar optical property results for the TiO_2_ coatings. According to [48], the *E*_g_^opt^ value for as-deposited TiO_2_ by EBE was equal to 3.84 eV. Taherniya et al. [48] also studied TiO_2_ films after annealing at 300, 450, and 600 °C, where the values of *E*_g_^opt^ slightly decreased to 3.83, 3.80, and 3.79 eV, respectively. These results are also in fair agreement with those reported by Yang et al. [6], who observed *E*_g_^opt^ values ranging from 3.81 to 3.92 eV for TiO_2_ films deposited by IBAD. Hasan et al. [49] reported an indirect optical bandgap of 3.39 eV for as-grown TiO_2_ films at room temperature, which is consistent with values reported in other studies [50–51]. However, it should be noted that the films in [49–51] were deposited using radio-frequency reactive and pulsed DC magnetron sputtering. These results indicate that the value of optical bandgap energy of the thin film coatings strongly depends on the conditions and deposition methods.

To complete analysis of the bandgap structure, the Urbach energy was evaluated based on the logarithmic plot of the absorption coefficient vs photon energy (Figure 5d–f). The slope of the linear dependence of ln α on *h*ν follows the exponential relation [46,52]:


[1] $$\alpha =\alpha_{0}\exp\left(\frac{h\nu }{E_{\text{u}}}\right),$$

where α_0_ is a constant and *E*_u_ is the Urbach energy.

After post-process annealing, *E*_u_ for the film deposited with conventional EBE method increased by almost four times from 0.22 to 0.87 eV (Figure 5d). The Urbach energies for as-deposited and annealed film deposited by IBAD (I_ibg_ = 3 A) were 0.25 and 0.24 eV, respectively. For coatings deposited with the additional *I*_ibg_ = 4 A, values of *E*_u_ were equal to 0.30 eV and 0.24 eV for as-deposited and annealed film, respectively. In the case of finely crystalline coatings, a higher Urbach energy value may indicate significant variation in the size and shape of the grains [53–55]. Based on the paper of Kim et al. [56], it can be assumed that when the film structure is well ordered, the Urbach energy value will be lower. Wiatrowski et al. [25] showed that the Urbach energies of TiO_2_ thin films with anatase and rutile phases, prepared by magnetron sputtering using continuous and pulsed gas flow processes were 0.17 and 0.24 eV, respectively. It was found that the lower value of *E*_u_ was obtained for the anatase thin film, which was directly related to the much more ordered structure of this layer, as found during the microstructure analysis. Table 3 summarizes the results of the optical properties.

**Table 3:** Optical properties of as-deposited and annealed coatings.^a^

	As-deposited			Annealed at 800 °C		
Sample	S0A	S3A	S4A	S0A	S3A	S4A

*T*_λ_ (%)	82.5	82.9	76.8	51	76.1	68.8
*E*_g_^opt^ (eV)	3.23	3.23	3.15	2.77	3.10	3.17
*E*_u_ (eV)	0.22	0.25	0.30	0.87	0.24	0.24

^a^*T*_λ_: optical transmission; λ_cutoff_: fundamental absorption edge; *E*_g_^opt^: optical bandgap energy; *E*_u_: Urbach energy.

**Figure 6:**
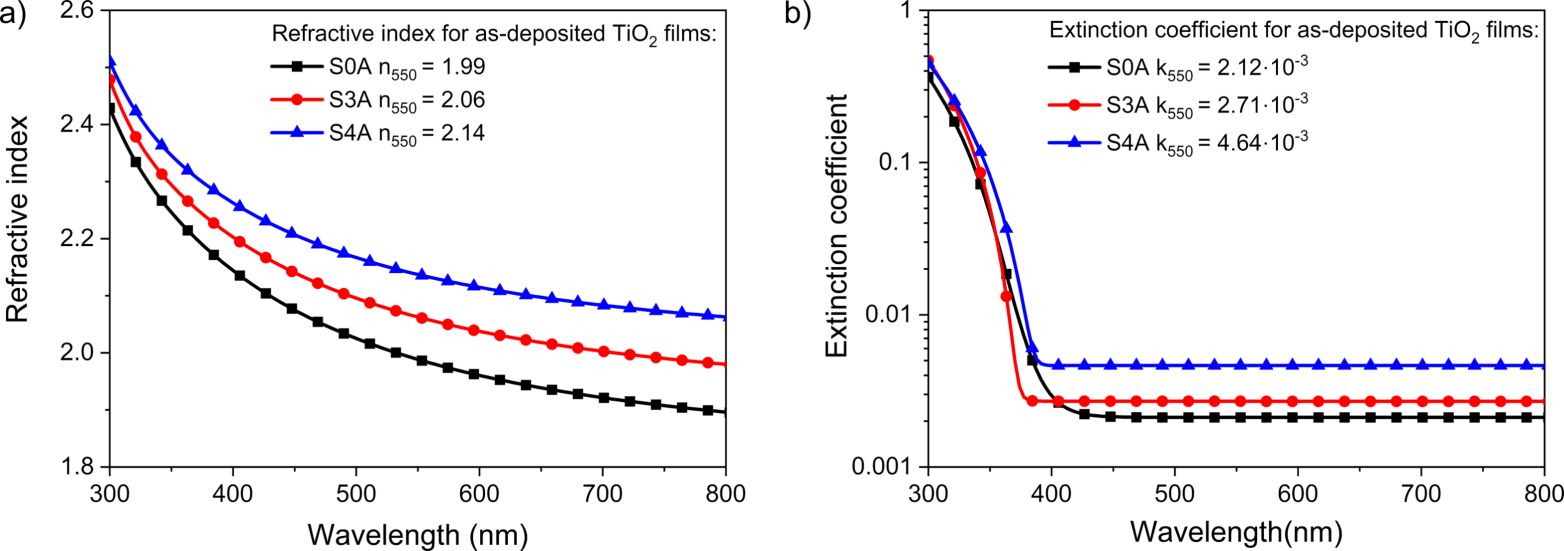
Comparison of (a) refractive index and (b) extinction coefficient of as-deposited TiO_2_ films.

For the prepared coatings, refractive index and extinction coefficient were determined using the reverse engineering method with the aid of the Scout software. For calculation of the dispersion curves, Sellmeier and O’Leary–Johnson–Lim [57] models were applied. The refractive index of the prepared films was in the range of 1.99 to 2.14 (Figure 6a). As the ion beam gun current increases, the amplitude of the interference also increases, which may indicate an increase in the refractive index of the prepared thin film coatings (Figure 4) and was proven in Figure 6a. The low value of the imaginary part of the refractive index, also known as extinction coefficient (Figure 6b), ranging from 2.12·10^−3^ to 4.64·10^−3^, indicates low light absorption in the studied thin films [58]. According to [6], TiO_2_ films prepared with the conventional EBE method had a porous structure with a refractive index of approximately 1.9. Selhofer et al. [59] reported that the value of the refractive index for TiO_2_ coatings deposited by reactive EBE (on unheated substrates) was in the range of 2.06 to 2.22, while in the case of substrates heated to 250 °C, the refractive index was 2.4.

Packing density (PD) and porosity (P) of both as-deposited and annealed coatings were determined using the real part of refractive index. Packing density was calculated according to the Clausius–Mossotti relation [60–61]:


[2] $$\text{PD}=\frac{\rho_{\text{f}}}{\rho_{\text{b}}}=\frac{\left(n_{\text{f}}^{2}-1\right)\left(n_{\text{b}}^{2}+1\right)}{\left(n_{\text{f}}^{2}+2\right)\left(n_{\text{b}}^{2}-1\right)},$$

where ρ_f_ and ρ_b_ represent the film and bulk densities of titania, respectively, while *n*_f_ and *n*_b_ denote the refractive indices of the TiO_2_ coatings and bulk material for amorphous titanium dioxide as 2.449 [43,61–62].

Porosity (P) was calculated based on the Equation 3 [63–64]:


[3] $$\text{P}\left(\%\right)=\left[1-\left(\frac{n_{\text{f}}^{2}-1}{n_{\text{b}}^{2}-1}\right)\right].$$

The highest packing density (0.87) and the lowest porosity (28.6%) were obtained for coatings deposited with the highest value of additional *I*_ibg_ = 4 A, while the opposite values were obtained for film deposited using the conventional EBE process (Table 4).

**Table 4:** Optical properties of as-deposited films.^a^

	As-deposited thin films		
	S0A	S3A	S4A

*n*_550_	1.99	2.06	2.14
*k*_550_	2.12·10^−3^	2.71·10^−3^	4.64·10^−3^
PD	0.79	0.83	0.87
P (%)	40.8	34.8	28.6

^a^*n*_550_: refractive index at λ = 550 nm; *k*_500_: extinction coefficient at λ = 550 nm; PD: packing density; P: porosity.

**Figure 7:**
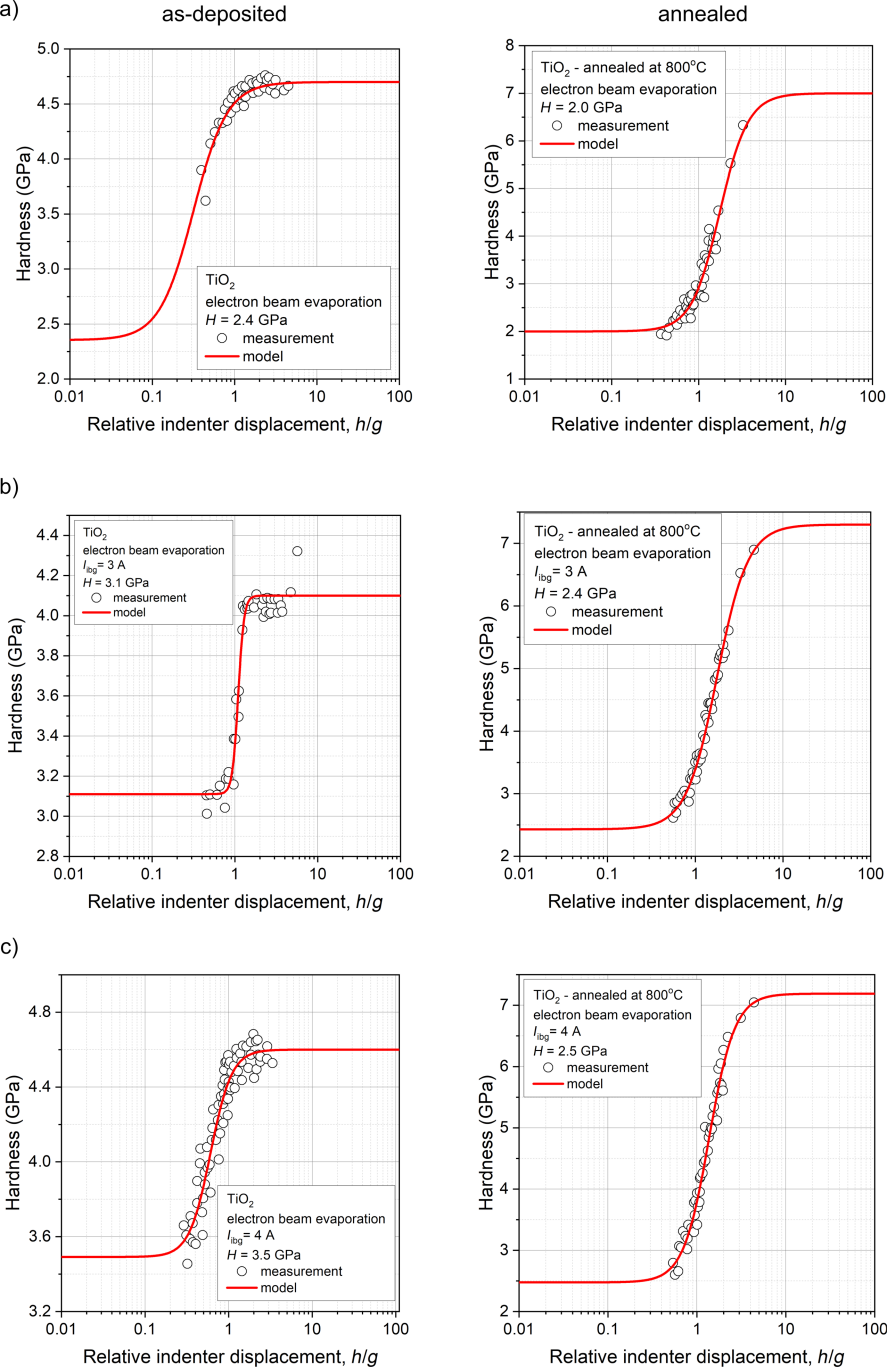
Hardness of as-deposited and annealed TiO_2_ thin films deposited by electron beam evaporation method (a) without and with IBAD with ion beam gun currents of (b) *I*_ibg_ = 3 A and (c) *I*_ibg_ = 4 A.

Annealing seems to play a key role in modifying the structure of the deposited TiO_2_ coating, as the grains tend to grow due to recrystallization and diffusion processes, leading to changes in density and structure. The formation of a double-layer coating, as observed in the cross-section scanning electron microscopy image (inset in Figure 4c) of the annealed TiO_2_ coating deposited with an additional *I*_ibg_ of 4 A, can be understood as the separation of grains into two distinct rows or layers. This phenomenon may be related to the deposition conditions and the post-process heat treatment of the titanium dioxide thin film. As the grains grow during annealing, the top row of grains may recrystallize differently from those near the substrate, forming a visible boundary. The separation between the two layers of grains may be related to the difference in the grain growth kinetics, which may be influenced by the annealing conditions. For optical coatings, amorphous, very smooth TiO_2_ layers with high refractive index and thickness up to 400 nm are required. Although the density and the refractive index of crystalline TiO_2_ are higher than those of the amorphous phase, it is often preferred in optical devices due to its lower surface roughness and light scattering [65]. Nanoindentation measurements (Figure 7) showed that the films deposited by EBE had a hardness of about 2.4 GPa. Increasing the ion gun current from 3 to 4 A caused an increase of hardness from 3.1 to 3.5 GPa. These measurements showed that the use of IBAD in the EBE process had a favorable effect on mechanical properties of prepared optical coatings. After additional annealing at 800 °C, the hardness of the titanium dioxide films decreased as compared to as-deposited films. The lowest hardness (2.0 GPa) was measured for the annealed coating without the use of an ion gun, while in the case of coatings deposited with the IBAD at 3 and 4 A, the hardness was equal to 2.4 and 2.5 GPa, respectively (Figure 7). To illustrate the impact of additional ion beam gun current on the hardness of thin films, the relationship between hardness and *I*_ibg_ for both as-deposited and annealed films is presented below in Figure 10a.

**Figure 8:**
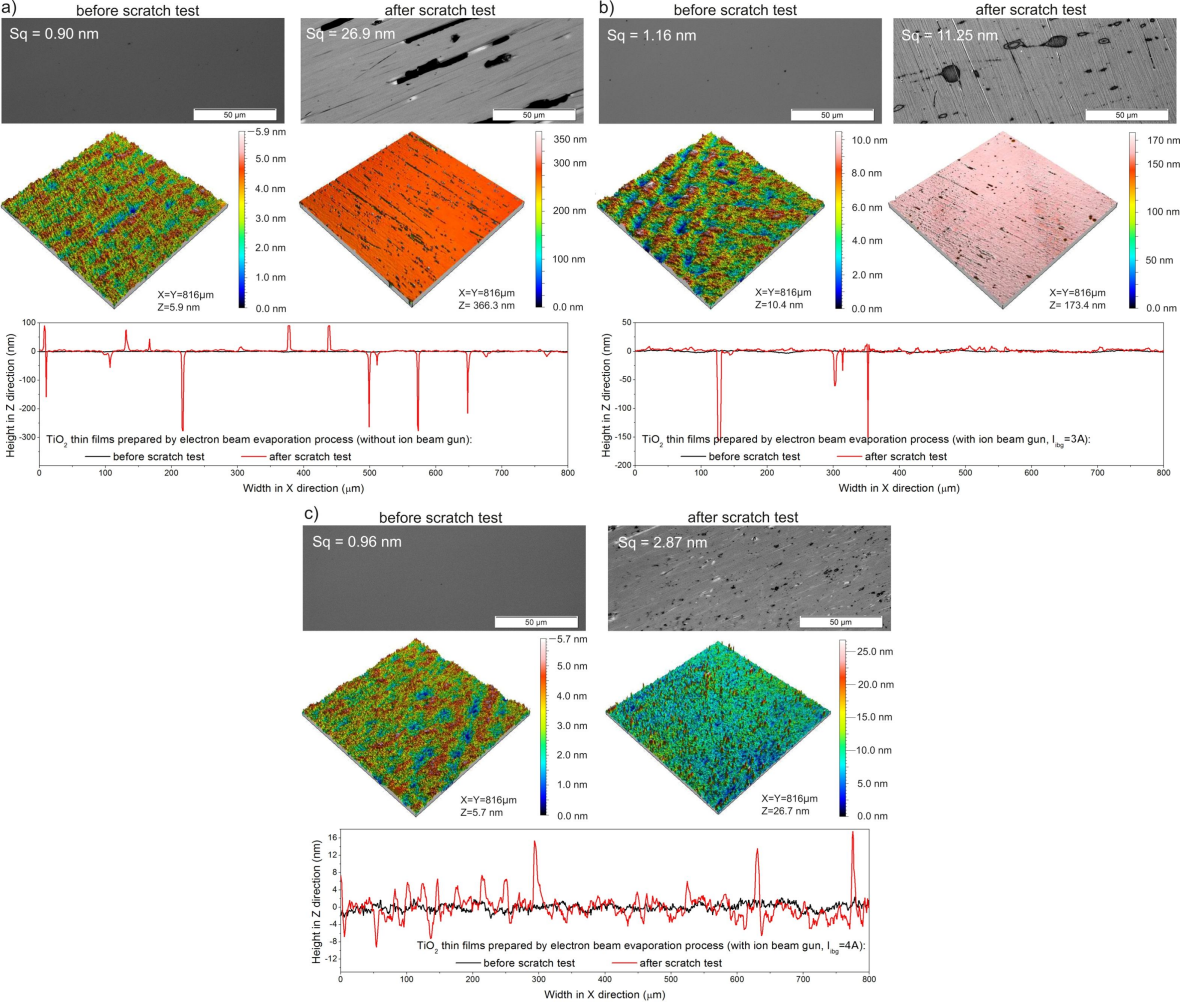
Results of surface topography measurements before and after abrasion tests of TiO_2_ thin films deposited by EBE (a) without and with IBAD with ion beam gun currents of (b) *I*_ibg_ = 3 A and (c) *I*_ibg_ = 4 A.

The results of abrasion resistance tests showed that the TiO_2_ coating deposited by the conventional EBE method had the worst abrasion resistance, which was also confirmed by tests with optical microscope and profilometer (Figure 8a). Based on the microscopic images, large scratches were observed. While measurements obtained by the optical profilometer showed that the scratches have a depth equal to the thickness of the thin film (Figure 8a). In the case of coatings prepared by the IBAD method with *I*_ibg_ = 3 A, the number of scratches formed after the tests was significantly lower, and the depth of the scratches was reduced by half (Figure 8b). In contrast, when an ion beam gun current of 4 A was applied, the scratches almost disappeared, and the cross-sectional surface profile showed that they were only a few nanometers deep (Figure 8c). The surface roughness of the coating deposited without the ion gun changed significantly after the abrasion test and was 26.9 nm, while for the films prepared with the gun with currents of 3 and 4 A, it was 11.3 and 2.9 nm, respectively. It can be assumed that the abrasion resistance of thin films produced by IBAD is due to the increased energy of ions reaching the substrates during deposition. In fact, this contributes to the increased density of the thin films. This increases the resistance to material removal and improves adhesion, which prevents delamination during abrasion. Moreover, the increased energy of ions during deposition led to the formation of a finer and more uniformly packed grain structure, which resulted in increased hardness and improved scratch resistance. Furthermore, the introduction of compressive residual stress could inhibit the initiation and propagation of cracks. Table 5 and Figure 10b below summarize the results of the mechanical properties tests.

**Table 5:** Summary of the results of measurements of the mechanical properties of TiO_2_ thin films deposited by EBE method without and with IBAD and after additional annealing at 800 °C.

	As-deposited			Annealed at 800 °C		
Sample	S0A	S3A	S4A	S0A	S3A	S4A

hardness (GPa)	2.4	3.1	3.5	2.0	2.4	2.5
Sq – before abrasion test (nm)	0.9	1.2	1.0	2.4	1.3	1.1
Sq – after abrasion test (nm)	26.9	11.3	2.9	2.3 (coating completely worn off)	119.6	88.2

**Figure 9:**
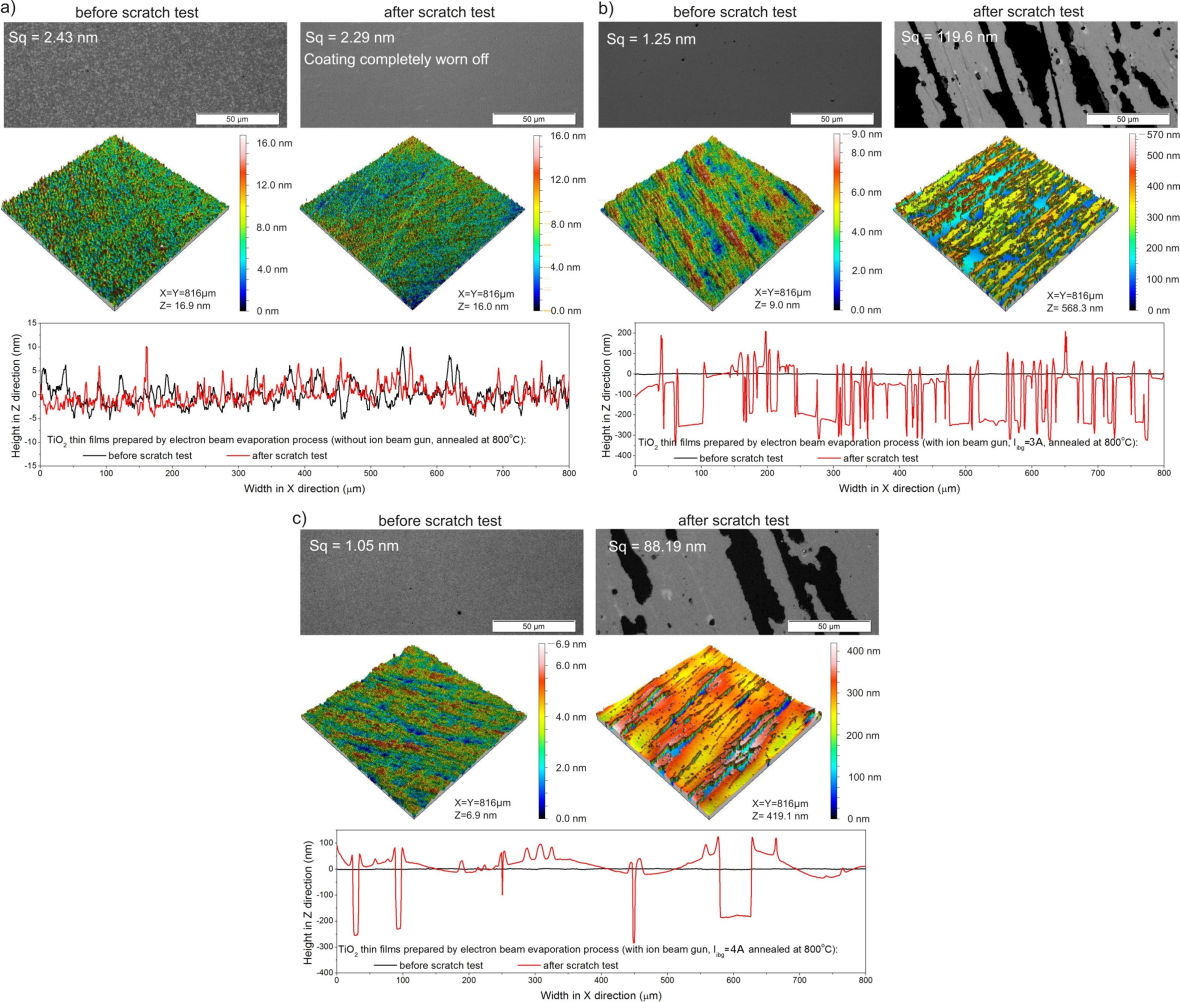
Results of surface topography measurements before and after abrasion tests of TiO_2_ thin films deposited by EBE (a) without and with IBAD with ion beam gun currents of (b) *I*_ibg_ = 3 A and (c) *I*_ibg_ = 4 A after additional annealing at 800 °C.

**Figure 10:**
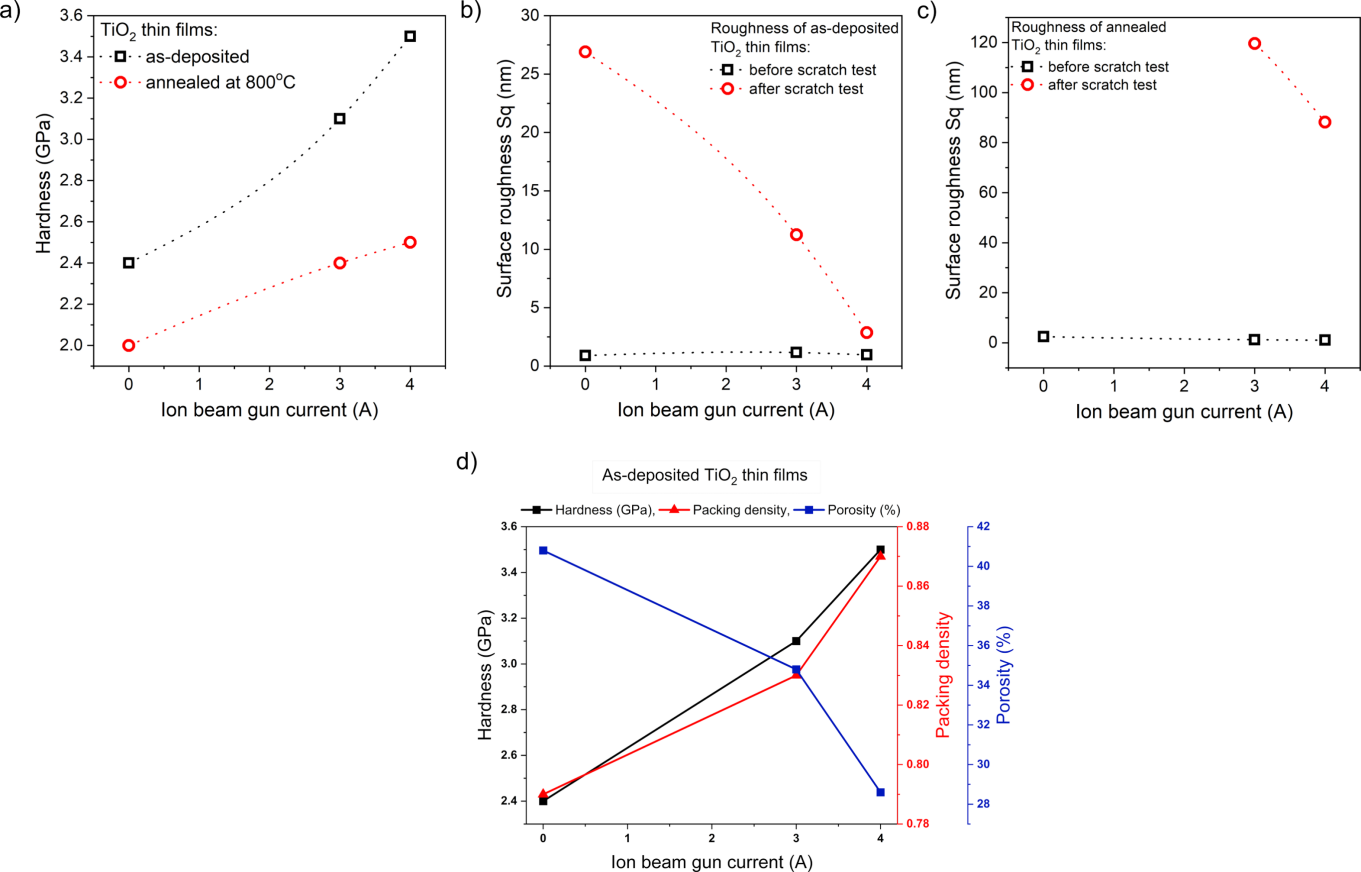
The dependence of (a) hardness and (b, c) surface roughness before and after abrasion tests on ion beam gun current for as-deposited and annealed thin films. (d) Hardness, packing density, and porosity as functions of the ion beam gun current in as-deposited films.

The results of surface topography measurements before and after abrasion tests for the annealed TiO_2_ thin films are included in Figure 9. Before the abrasion test, all coatings were homogeneous and had low roughness values from 2.0 to 2.5 nm. However, after the steel wool tests, the film deposited without the ion gun and annealed at 800 °C was completely rubbed off from the surface of the substrate (Figure 9a). Coatings deposited with IBAD were scratched quite significantly, but the thin film was not completely removed from the surface (Figure 9b). In the case of the coating deposited with the ion gun current of 3 A, the depth of the scratches was equal to the thickness of the thin film, and about half of the coating was removed from the substrate. Increasing the ion beam gun current resulted in scratches still having a depth equal to the film thickness, but only about 30% of the coating was removed from the substrate (Figure 9c). The results of the mechanical properties after annealing are shown in Table 5 and Figure 10c.

Based on the SEM images of the surface morphology and cross sections of the thin films, along with the mechanical results (Table 5), Thornton’s model [66–67] can be referred to. For the EBE-deposited thin films, the coatings were assigned into the less dense Zone 1 or Zone T (transition), where low adatom mobility resulted in a coarse-grained columnar structure with voids between the grains, clearly observable in the annealed films (Figure 3a). This structural characteristic explains the observed lower hardness and abrasion resistance (Figure 10). In contrast, for the thin films prepared with additional ion beam gun current, the SEM images indicate a transition from the less dense Zone 1 or Zone T to a more compact Zone 2 microstructure (Figure 3b,c). The use of additional *I*_ibg_, which increases the total energy of the particles reaching the substrate through ion bombardment, leads to densification and enhanced mechanical properties of the films. This phenomenon explains the observed increase in hardness and wear resistance (Figure 10a,b). Furthermore, as shown in Figure 10d, a strong correlation is evident: As the packing density increases, the hardness increases while porosity simultaneously decreases.

## Conclusion

In the present work, electron beam evaporation and ion beam-assisted deposition were used to prepare titanium dioxide thin films. Additionally, post-process annealing at 800 °C was applied to study its effect on the properties of the prepared films. This method is very promising, as it allows for precise control over the growth process and makes it possible to adjust the properties of the titania thin films to achieve the desired properties. Although this method is well known, as is titanium dioxide, it is very difficult to find research that provides a comprehensive discussion and comparison of the impact of IBAD on the conventional EBE method regarding structural, optical, mechanical, or tribological properties.

A comparison of XRD patterns for deposited and annealed TiO_2_ thin films prepared by EBE and IBAD methods reveals a significant structural transformation from the amorphous to the anatase phase after annealing. The results show that the use of ion beam-assisted deposition during EBE did not significantly affect the prepared structure but significantly affected the size of the crystallites, with higher ion gun currents resulting in smaller crystallites in the range of 30.8 to 43.5 nm. Additionally, Raman spectroscopy confirmed that only the anatase phase was present after annealing at 800 °C. SEM images reveal that post-process annealing significantly affects the morphology of TiO_2_ thin films, highlighting the differences between coatings prepared with and without additional IBAD. All as-prepared coatings exhibit a smooth, columnar morphology with small grains. Moreover, the SEM images show that after annealing, the TiO_2_ film deposited by the EBE method formed large grains approximately 100 nm in size, which clustered into agglomerates with voids. This is visible in the cross-section images as a coarse-grained structure with grain sizes ranging from 160 to 350 nm. In comparison, coatings deposited with IBAD have more compact, rounded grains, with sizes decreasing from 58 to 45 nm as the ion beam gun current increases from 3 to 4 A. Annealing further modifies the cross-sectional morphology of the 3 A films, resulting in elongated grains measuring from 60 to 120 nm. In contrast, the 4 A films exhibit a bilayer structure with distinct layers, where grain widths range from 90 to 115 nm. The light transmission spectra of as-deposited TiO_2_ thin films reveal high transparency in the visible range with values between 77% and 83%, depending on the value of ion beam gun current. It is worth to notice, that films deposited with *I*_ibg_ = 3 A achieved the highest transparency at 82.9%, while those with deposited with *I*_ibg_ = 4 A exhibited slightly lower transparency at 76.8%. Post-process annealing lead to a significant decrease in the transparency level to 51% for film EBE-deposited films. In turn, in the case of coatings prepared by the IBAD method, post-process annealing had little effect on the level of transparency, which decreased by about 6–8%. The refractive index of the prepared films was in the range from 1.99 to 2.14. An increase in the value of ion beam gun current led to a higher amplitude of interferences, resulting in an increase in the refractive index. In addition, the low values of the extinction coefficient (from 2.12·10^−3^ to 4.64·10^−3^) indicated minimal light absorption in the tested films. The results of the nanoindentation studies showed that the hardness of thin films deposited by the conventional EBE method was significantly enhanced by using ion beam-assisted deposition, with hardness values increasing from 2.4 to 3.5 GPa. However, post-process annealing lead to a decrease in hardness values, particularly in coatings deposited without additional *I*_ibg_, highlighting the impact of processing conditions on material properties. Abrasion resistance tests showed that the TiO_2_ film deposited using the EBE method was the least abrasion-resistant compared to the IBAD films.

To conclude, the use of additional ion-beam assistance during thin film deposition by the EBE method significantly increases the density and structural uniformity of the films, advancing them from the less dense Zone 1 to the more compact microstructure of Zone 2. This transition results in improved mechanical properties, including increased hardness and wear resistance, highlighting the key role of IBAD in tailoring the desired properties of TiO_2_ coatings.

## Data Availability

Data generated and analyzed during this study is available from the corresponding author upon reasonable request.

## References

[1] Duyar Ö, Placido F, Zafer Durusoy H (2008). J Phys D: Appl Phys.

[2] Lin S-S, Chen S-C, Hung Y-H (2009). Ceram Int.

[3] Lu Z, Jiang X, Zhou B, Wu X, Lu L (2011). Appl Surf Sci.

[4] Hsu S-C, Hong J-Y, Chen C-L, Chen S-C, Zhen J-H, Hsieh W-P, Chen Y-Y, Chuang T-H (2021). Appl Surf Sci.

[5] Lee S-H, Choi M, Jung Y-I, Sim S-J, Moon J-K, Choi J, Kim S (2022). Thin Solid Films.

[6] Yang C, Fan H, Xi Y, Chen J, Li Z (2008). Appl Surf Sci.

[7] Chen H-C, Lu Y-R, Chang C-H (2024). Thin Solid Films.

[8] Jaing C-C, Chen H-C, Lee C-C (2009). Opt Rev.

[9] Li Y-Q, Wang H-Q, Wang W-Y, Yu Z-N, Liu H-S, Jin G (2012). Acta Mech Sin.

[10] Smidt F A (1990). Int Mater Rev.

[11] Rauschenbach B (2002). Vacuum.

[12] Placido F, Gibson D (2010). Chin Opt Lett.

[13] Fulton M L (1994). Proc SPIE.

[14] Mansilla C (2009). Solid State Sci.

[15] Almaev A V, Yakovlev N N, Kushnarev B O, Kopyev V V, Novikov V A, Zinoviev M M, Yudin N N, Podzivalov S N, Erzakova N N, Chikiryaka A V (2022). Coatings.

[16] Rasim Mohammed H, Mohammed Hadi Shinen D (2023). Mater Today: Proc.

[17] Khan A, Gaikwad M A, Kim J H, Kadam A (2024). Tungsten.

[18] Khan A, Kadam A V (2024). Tailoring TiO2 Films: The Path to Superior Electrochromic Performance. Titanium-Based Alloys – Characteristics and Applications.

[19] Žerjav G, Žižek K, Zavašnik J, Pintar A (2022). J Environ Chem Eng.

[20] Rafieian D, Ogieglo W, Savenije T, Lammertink R G H (2015). AIP Adv.

[21] Hui B, Fu X, Gibson D, Child D, Song S, Fleming L, Rutins G, Chu H O, Clark C, Reid S (2018). Coatings.

[22] Macleod H A (2001). Thin-Film Optical Filters.

[23] Obstarczyk A, Mazur M, Kaczmarek D, Domaradzki J, Wojcieszak D, Grobelny M, Kalisz M (2020). Thin Solid Films.

[24] Obstarczyk A, Kaczmarek D, Mazur M, Wojcieszak D, Domaradzki J, Kotwica T, Morgiel J (2019). J Mater Sci: Mater Electron.

[25] Wiatrowski A, Mazur M, Obstarczyk A, Wojcieszak D, Kaczmarek D, Morgiel J, Gibson D (2018). Coatings.

[26] Oliver W C, Pharr G M (1992). J Mater Res.

[27] Mazur M, Wojcieszak D, Kaczmarek D, Domaradzki J, Song S, Gibson D, Placido F, Mazur P, Kalisz M, Poniedzialek A (2016). Appl Surf Sci.

[R28] 28ISO/TC 172/SC 7/WG 3 N30 Standard, Spectacle lenses – Test method for abrasion resistance; 1998.

[29] Blacker R, Bohling D, Coda M, Kolosey M (2000). Development of Intrinsically Conductive Antireflection Coatings for the Ophthalmic Industry. 43rd Annual Technical Conference Proceedings, Society of Vacuum Coaters.

[30] Mergel D, Buschendorf D, Eggert S, Grammes R, Samset B (2000). Thin Solid Films.

[31] Oliver J B, Kupinski P, Rigatti A L, Schmid A W, Lambropoulos J C, Papernov S, Kozlov A, Spaulding J, Sadowski D, Chrzan Z R (2011). Appl Opt.

[32] Soussi A, Ait Hssi A, Boujnah M, Boulkadat L, Abouabassi K, Asbayou A, Elfanaoui A, Markazi R, Ihlal A, Bouabid K (2021). J Electron Mater.

[33] Lotgering F K (1959). J Inorg Nucl Chem.

[34] Brosnan K H, Messing G L, Meyer R J, Vaudin M D (2006). J Am Ceram Soc.

[35] Furushima R, Tanaka S, Kato Z, Uematsu K (2010). J Ceram Soc Jpn.

[36] Nair P B, Justinvictor V B, Daniel G P, Joy K, Ramakrishnan V, Thomas P V (2011). Appl Surf Sci.

[37] Jena S, Tokas R B, Misal J S, Rao K D, Udupa D V, Thakur S, Sahoo N K (2015). Thin Solid Films.

[38] Ekoi E J, Gowen A, Dorrepaal R, Dowling D P (2019). Results Phys.

[39] Ohsaka T, Izumi F, Fujiki Y (1978). J Raman Spectrosc.

[40] Porto S P S, Fleury P A, Damen T C (1967). Phys Rev.

[41] Kadam R M, Rajeswari B, Sengupta A, Achary S N, Kshirsagar R J, Natarajan V (2015). Spectrochim Acta, Part A.

[42] Dave V, Gupta H O, Chandra R (2014). Appl Surf Sci.

[43] Jolivet A, Labbé C, Frilay C, Debieu O, Marie P, Horcholle B, Lemarié F, Portier X, Grygiel C, Duprey S (2023). Appl Surf Sci.

[44] Rahimi N, Pax R A, Gray E M (2016). Prog Solid State Chem.

[45] Nezar S, Saoula N, Sali S, Faiz M, Mekki M, Laoufi N A, Tabet N (2017). Appl Surf Sci.

[46] Dejam L, Sabbaghzadeh J, Ghaderi A, Solaymani S, Matos R S, Țălu Ș, da Fonseca Filho H D, Hossein Sari A, Kiani H, Hossein Salehi Shayegan A (2023). Sci Rep.

[47] Obstarczyk A, Mańkowska E, Weichbrodt W, Kapuścik P, Kijaszek W, Mazur M (2024). Opto-Electron Rev.

[48] Taherniya A, Raoufi D (2016). Semicond Sci Technol.

[49] Hasan M M, Haseeb A S M A, Saidur R, Masjuki H H, Hamdi M (2010). Opt Mater (Amsterdam, Neth).

[50] Wang T M, Zheng S K, Hao W C, Wang C (2002). Surf Coat Technol.

[51] Karuppasamy A, Subrahmanyam A (2007). J Appl Phys.

[52] Brodsky M H (1985). Amorphous Semiconductors.

[53] Tang H, Lévy F, Berger H, Schmid P E (1995). Phys Rev B.

[54] Domaradzki J, Kaczmarek D, Prociow E L, Borkowska A, Schmeisser D, Beuckert G (2006). Thin Solid Films.

[55] Domaradzki J (2010). Powłoki optyczne na bazie TiO2.

[56] Kim D, Ahn S, Kwon H (2006). Thin Solid Films.

[57] O’Leary S K, Johnson S R, Lim P K (1997). J Appl Phys.

[58] Mazur M, Domaradzki J, Wojcieszak D (2014). Bull Pol Acad Sci: Tech Sci.

[59] Selhofer H, Ritter E, Linsbod R (2002). Appl Opt.

[60] Dave V, Dubey P, Gupta H O, Chandra R (2013). Thin Solid Films.

[61] Mazur M (2017). Opt Mater (Amsterdam, Neth).

[62] Bradley J D B, Evans C C, Choy J T, Reshef O, Deotare P B, Parsy F, Phillips K C, Lončar M, Mazur E (2012). Opt Express.

[63] Kermadi S, Agoudjil N, Sali S, Zougar L, Boumaour M, Broch L, En Naciri A, Placido F (2015). Spectrochim Acta, Part A.

[64] Subramanian M, Vijayalakshmi S, Venkataraj S, Jayavel R (2008). Thin Solid Films.

[65] Ratzsch S, Kley E-B, Tünnermann A, Szeghalmi A (2015). Nanotechnology.

[66] Thornton J A (1974). J Vac Sci Technol (N Y, NY, U S).

[67] Anders A (2010). Thin Solid Films.

